# Designing for the post-pandemic era: Trends, focuses, and strategies learned from architectural competitions based on a text analysis

**DOI:** 10.3389/fpubh.2022.1084562

**Published:** 2022-12-07

**Authors:** Pei Han, Lingju Wang, Yufei Song, Xi Zheng

**Affiliations:** ^1^Department of Architecture, School of Architecture and Civil Engineering, Harbin University of Science and Technology, Harbin, China; ^2^Harbin Institute of Technology Architectural Design and Research Co., Harbin, China

**Keywords:** COVID-19, architectural competitions, text mining analysis, statistical analysis, design strategies, social focuses, design trends

## Abstract

The COVID-19 pandemic has made the built environment an important source of prevention and control, architects and scholars have thus been seeking countermeasures since the beginning of the outbreak. As design and construction cycles are long, only a few completed cases and evidence-based studies are available for reference. However, massive architectural competition works have emerged, which always been the soil for discussion and practice of cutting-edge design issues. These contain a vast number of ideas for solutions from various design dimensions—including cities, buildings, and facilities—and provide a great deal of materials worth analyzing and summarizing. Therefore, the exploration of competitions will provide us with public health intervention directions, strategies and a rethinking of the built environment. Using a text-mining approach, we analyzed 558 winning entries in architectural competitions related to the pandemic response, exploring specific issues, populations involved, coping strategies, and trends that emerged as the pandemic evolved. Our results show that the strategies proposed can be grouped into 17 keywords, with modularization being the most frequent strategy and related strategies like rapid assembly, flexible space, etc. are also took a significant percentage of the use. Further, we explored the technical orientation, year, territory, target groups, and target problems of the works which lead to a series of cross-comparison relationships. The results indicate that indirect impacts caused by the pandemic gained more attention and flexible Solutions were used more often highlighted the consensus when adapting to the uncertainties. The focus on the spiritual dimension is increasing year by year reflected the spiritual influences were gaining traction and the indirect impacts gradually showed up over time. The research will provide a strategy reference for the design response to the pandemic, as well as help understand the influence and significance of social factors behind the divergence of issue focuses and strategic tendency in different regions and times.

## Introduction

The coronavirus disease 2019 (COVID-19) outbreak has had a massive impact on all of society and has triggered a major public health crisis (1). Despite the implementation of measures such as social distancing, lockdowns, border closures, and human tracing having effectively controlled the spread of COVID-19 (2), there were concurrently multiple secondary impacts (3, 4), such as psychological effects (5, 6) and changes to physical activities, functions, and quality of life (7).

The transmission of infectious diseases among the populace is related to epidemiology and even more closely to the built environment. The intersection of the two fields was noticed as early as the Middle Ages when controlling environmental factors helped bring an end to the bubonic plague (Black Death) in the 14^th^ century. This launched related research on the built environment as an intervention in public health (8). The cholera outbreak in London in the 19^th^ century brought awareness of the geographic linkages of the disease (9), which led to changes in the approach to urban planning. These included the creation of larger public spaces between buildings and more organized layouts, governance of dirty and overcrowded neighborhoods, and introduction of additional parks and green spaces in the city center (10). The urban design of Paris incorporated extensive amounts of lengthy streets and open spaces to establish a well-developed infrastructure for the sewage system (11). In the 1950s, building designs incorporated terraces, balconies, and flat roofs to curb the spread of tuberculosis and other respiratory diseases (12).

Thus, public health issues have major impacts on urban planning, design, and development, and the related crises have resulted in the restructuring and optimization of urban spaces (13). These historical experiences of integrating the built environment and public health fully demonstrate that urban design is an integral factor in improving public health (14, 15) and plays a critical role in the prevention of disease outbreaks; the control, isolation, and mitigation of pandemics; and the formulation of countermeasures after such events (16).

The COVID-19 pandemic has persisted for nearly 3 years. During this period, there have been enthusiastic discussions in academia and the industrial sector, leading to diverse perspectives on related issues and countermeasures. These include the influence of different environments on wellbeing during the COVID-19 period (17, 18), the need to develop a resilient urban system of the future (19), and a paradigm shift toward the built environment to consider the effects of pandemics and informatization (20). However, building and urban development involve lengthy construction cycles, and evidence-based research on optimizing the built environment for pandemic conditions is limited. Most existing research aimed to uncover the principles and discuss the related impacts (21–25). However, designers are practitioners and work mostly based on their accumulated experience and the relevant specifications of their current projects. The impact of the pandemic on design goals and methods is enormous, and it will take years to develop new design guidelines based on experience and post-occupancy evaluations (POE) of completed works. In response to the pandemic, a large number of new construction or renovation optimization projects have emerged. While the final structures will be used for decades after their completion, these programs often involve short-term cycle and heavy design tasks, leaving little opportunity for architects to explore existing social needs and solutions. Nevertheless, unearthing existing needs and responding to them is an important challenge for the industry today that directly affects the long-term influence of the built environment on public health in the post-pandemic era.

In the academic and industry sectors, architectural design competitions have always facilitated thought experiments and free expression of ideas on social issues. Architectural design in a broad sense encompasses a wide range of content, from urban planning to building design to landscape and interiors, all within the larger architectural context (26); in short, the built environment, and therefore the architectural design competition entries, often include a discussion of all aspects of the built environment. Previous research on this topic often focused on the presentation and analysis of one or several such competitions, such as compiling the competition works into publications to showcase the results, discussing the issues faced during the process of the competitions (27–30), performing comparative analyzes of various series of competitions (31–33), and discussing the strategies derived from specific designs presented in the competitions (34–37). Similarly, there was a flourishing of ideas on various issues related to the pandemic after its outbreak. There were numerous discussions on the use of design strategies for the built environment as interventions for the pandemic, as well as thoughts and reflections on the pandemic from various perspectives. Architects generated a large number of cases and refined design methods in a short period of time in response to the changes in the environment. Cumulatively, these are valuable resources for design practices and constitute a good opportunity for comprehending the variations in focus across different geographic regions and over time. By analyzing these works we hope to obtain a series of inspiring solutions for architects and decision makers, but also to explore the commonalities and differences and the reasons behind them.

Following from the above, the issues being examined in this study were as follows:

**RQ1:** What specific social issues did the competition works focus on as a result of the pandemic? What were the proposed solutions to those issues?
**RQ2:** Have the attitudes and focus on social issues changed over time with the progression of the pandemic? Have the strategies proposed for resolving the issues been revised accordingly?
**RQ3:** Do the focus and proposed strategies differ from a geographic perspective?

## Materials and methods

### Data sources and processing

To gather as many competition works related to the pandemic as possible, the research team conducted searches at two levels: competitions and design works. Given that the COVID-19 infection first appeared at the end of 2019, we searched within the timeframe of 2020 until August 2022. This would include all architectural competitions held in 2020–2022. First, the team screened competition themes posted on websites within and outside China pertaining to renowned architectural design competitions. This included more than 10 websites such as ArchDaily/Architecture Competitions, Arc race, Archinect, Bustler, Archi Competition, and the competitions blog, which are specialized websites that collect and publish information on architectural design competitions or professional websites that share winning works. The relevant competition information was found through searches using keywords such as “pandemic” and “COVID-19” with the winning works being directly added to the database.

Second, the massive changes brought on by the pandemic throughout society led to many design works intending to deal with and solve the related problems. The award-winning works were manually identified according to the well-known series of competitions that have been held for many years. The pandemic was not the sole competition theme being considered; any design work that dealt with the topic was collected. Finally, general Internet searches were conducted using keywords such as “pandemic,” “COVID-19,” and “competition” to supplement the database with a small number of competitions and works not found in earlier searches.

### Review data collection

**Figure 1:**
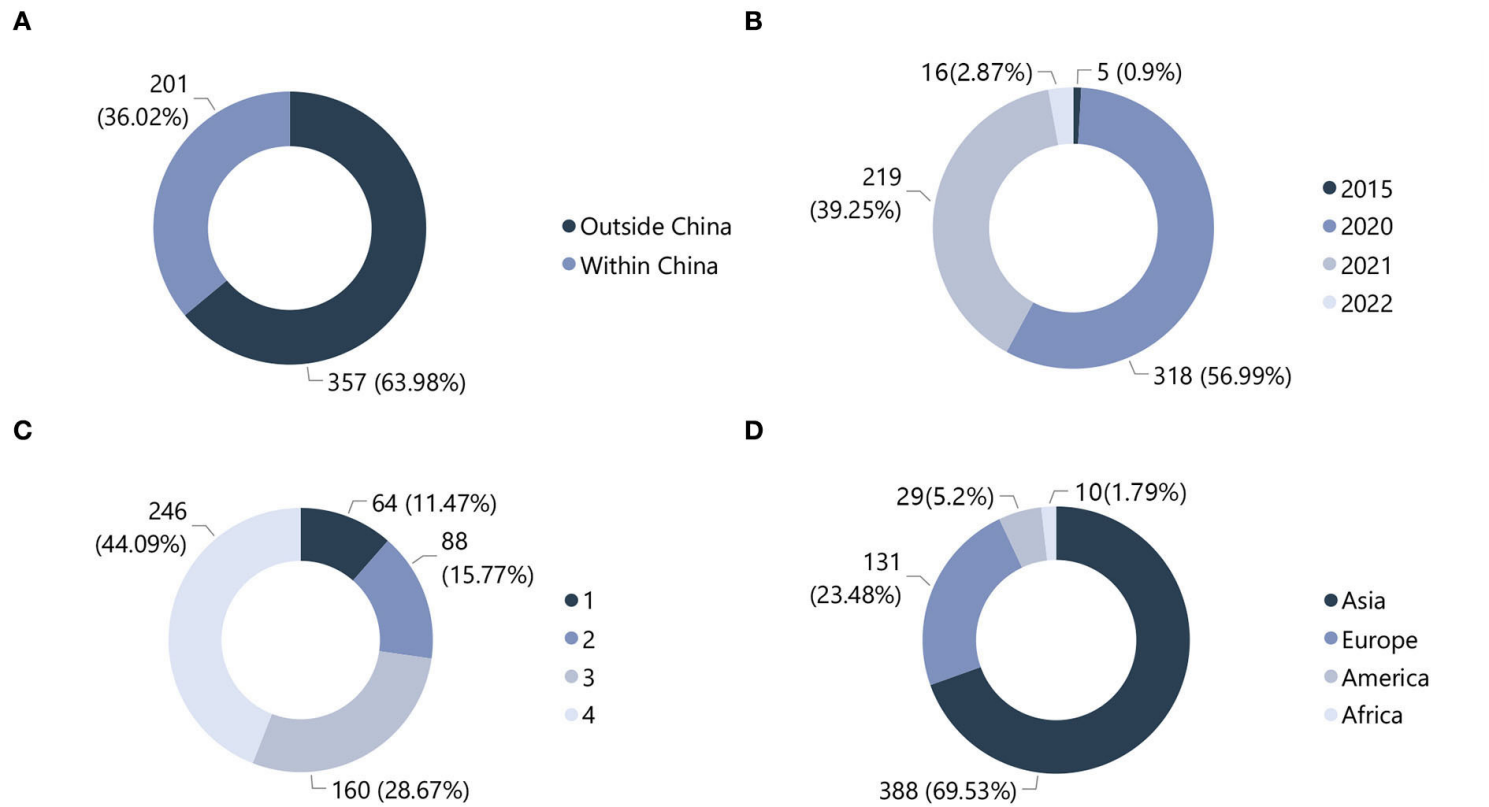
Distribution of collected competition works. **(A)** Territory. **(B)** Year. **(C)** Award level. **(D)** Country.

We compiled 553 winning works from 56 competitions (Figure 1). Among them, 33 competitions had themes that were clearly limited to COVID-19, and the remaining 23 had winning works produced within this scope. There were 20 and 36 competitions hosted within and outside China, respectively. From the annual distribution, in 2020, 2021, and 2022, there were 318, 219, and 16 works, respectively.

A search of the related competitions led to the discovery of five works in 2015 related to the pandemic theme. These works focused on infectious diseases such as cholera and Ebola, which broke out in Africa. Considering that works with similar concerns at different times could provide useful comparisons, these were also incorporated into the overall database of winning works, giving a total of 558 works. Of all the studied works, 201 and 357 were from within and outside China, respectively. A categorization was made according to the geographic location of the competition organizers. Asia had the highest number of works at 388, followed by Europe (131), America (29), and Africa (10).

The various competitions had different ways of ranking the winning works. Thus, in addition to the explicit first, second, and third prizes, which were recorded as Award Levels 1–3, respectively, a fourth category of Award Level 4 was added to include works with excellence awards and shortlisted finalists.

A preliminary database was prepared by sorting out the recorded information on the name, theme, year, and other textual information on the competitions, as well as the regions the competition winners were from, their award levels, and descriptions of their works.

## Methods

The four steps of graphic language conversion, text mining, data coding, and text analysis were applied sequentially to transform the drawing works from graphic to textual languages for the purpose of statistical analysis. After key information was extracted from large amounts of textual data, the unstructured textual information was encoded before the meaning and lessons learned were obtained. These constituted the four portions of the research contents.

An important preliminary task was to convert and break down the competition drawings into the textual language required for subsequent research. Some works had textual descriptions provided on the winning pages or drawings. After a manual inspection to determine that all information required for the study was covered, the texts were directly extracted and entered into the database. The tasks of image recognition and textual description had to be manually completed for works with no or incomplete information provided. All researchers involved are professionals, and accuracy during conversion was ensured by their ability to parse and extract the design ideas and key information from the drawings. In addition to the corpus required for text mining after conversion of the design descriptions, other details such as the target population and selected building types were included separately in the database.

The contents of text mining in this study were divided into two parts: competition themes and introduction of the works. Further exploring the themes set by the competition organizers helped us understand the focus and guiding direction of these institutions or organizations in the context of the pandemic. Delving into the introduction of the works also allowed us to grasp the observations and responses that were prominent in the design perspectives. In other words, the first part pertained to observations at the social level, and the second part related to ideas at the individual level.

Text mining as a mainstream methodology has a wide range of applications in many fields (38–40), which involves techniques such as distribution analysis, clustering, trend analysis, and association rules, etc., (41). In this study, the NLPIR Big Data Semantic Intelligent Analysis Platform 1.0.0.1 (42) and WordArt (https://wordart.com/) were used for text mining and visualization of the results, respectively. First, all the competition topics and work descriptions with the pandemic theme were compiled into one document to form a source folder for the corpus. After accessing the NLPIR platform and launching the “New word discovery” tab, the corpus was imported for the extraction of new words and keywords (43). The extracted results were entered into a user dictionary, together with labeled keywords that were manually summarized. Next, the user dictionary was imported to the “Batch word segmentation” tab for word segmentation of the corpus. Following this step, the common stopword list was imported to the “Language statistics” tab for compiling the statistics on word frequency. The meaningful and meaningless words were screened and categorized accordingly, with the latter placed in the stopword list. The statistics on word frequencies were then compiled again. The entire process was repeated to increase the accuracy of the word frequency data. After one round of manual screening, the word frequency data were entered into WordArt to generate word clouds.

The word frequency results obtained through text mining were classified into two groups: issues and solutions. These formed the main basis for data encoding. The core focus of this study was to identify the issues that the competition works highlighted in the context of the pandemic and the proposed solutions. The first step was coding the issues toward which the works were oriented. Descriptions of the issues gathered during the previous step of graphic language conversion were summarized into five primary and seven secondary categories. The primary categories were (1) original building and space form non-adapted to the normalization of pandemics, (2) spiritual needs in the context of a pandemic, (3) medical resource supply shortage, (4) medical building optimization, and (5) unreasonable resettlement of floating personnel.

Under Category (1) were two secondary categories: unreasonable function and organization of human living space in a pandemic situation and unreasonable urban public space in a pandemic situation. Under Category (2) were two secondary categories as well: commemoration of people and events related to the pandemic and restoration of the psychological impact of the pandemic. After extraction from the converted text, the strategies of the competition works in response to the pandemic were coded to form 17 strategy labels. These covered the design contents highlighted by the high-frequency words. There was also a small number of labels that were not high-frequency words, some of which could be grouped under similar keywords according to their contents.

A work might correspond to more than one strategy label owing to the complexity of the works and the various foci under the scope. For example, Work No. 275 integrated eight strategies to enhance the adaptability of urban public spaces as its response to the pandemic. After the coding was completed, information on the works, including the year, territory (within or outside China), continent, award level, technical orientation, and target group, was treated as the input variables. Following this step, the graphic language was completely converted into label information, and the complete database of 558 works was finally formed for subsequent analysis.

The statistics and description of the data were of great significance in this study. We used these numbers and proportions to understand the designers' thoughts and solutions to the pandemic, as well as the common and evolutionary characteristics. IBM SPSS Statistics 26 software was used to conduct statistical computations of the frequency and percentage of each variable in the database. After the chi-square test, pair-wise cross-tabulation analysis was performed on those variables with significant differences to determine the variations in data under different conditions of variable classifications.

## Results

### Results of text mining

The acquired data were divided into two parts: information on the competition themes and that on drawing works information. After NLPIR and manual processing, 29 new words and 92 keywords were obtained for information on the competition themes, and 36 new words and 89 keywords were obtained for drawing works information. The new words and keywords were imported into the word segmentation dictionary to obtain more accurate results for word segmentation, leading to the statistical results for single and binary word frequencies. There were 39 single word frequencies on competition themes, and 79 single word frequencies and 20 groups of binary word frequencies for drawing works information. Table 1 shows the top 30 words with the highest frequency extracted from the themes and works.

**Table 1:** High frequency vocabulary of competition themes and works.

**Themes** **word frequency**		**Themes** **key words**			**Works** **word frequency**		**Works** **key words**		
**Words**	**Freq**.	**Words**	**Weight**	**Freq**.	**Words**	**Freq**.	**Words**	**Weight**	**Freq**.
City	123	Complex system	19.28	9	Modularization	79	City	73.02	124
Society	68	Sports	15.29	22	Unit	60	Community	68.99	128
Epidemic situation	65	Quarantine	14.41	15	Public space	40	Modularization	45.41	79
Community	58	Body building	14.23	24	Medical treatment	31	Public space	30.15	40
Health	51	Health	13.71	52	Pandemic era	31	Vertical	25.79	34
Health	22	Public space	12.81	13	Roof	30	Medical treatment	24.82	32
Sports	21	Fitness products	12.64	4	Device	30	Roof	22.31	30
Body building	20	Comfort	12.48	5	Elastic	26	Move	21.84	43
Architect	17	Memorial hall	10.92	12	Hospital	25	Flat and pandemic combination	21.11	4
Epidemic era	16	Facade system	10.89	5	Streamline	25	Street	20.66	23
Certainty	13	Education	10.56	10	Facilities	24	Hospital	19.99	25
Public space	13	Medical treatment	10.55	12	Security	23	Ecology	19.63	23
Medical treatment	12	Park city	10.36	3	Street	23	Elastic	19.05	27
Ecology	12	Memorial device	9.31	3	Ecology	23	Improve living quality	17.47	11
Family	12	Social distance	9.25	6	Health	21	Green	16.47	15
Memorial hall	12	Daily life	8.78	11	Socializing	18	Health	16.35	44
Daily life	11	High-rise buildings	8.76	6	Toughness	18	Sharing	16.25	23
Green	10	Green	8.69	10	Exchange	18	Landscape	16.16	20
Density	10	Density	8.69	10	Vertical	16	Social distance	16	16
Normal	10	Multidimensional	8.64	3	Green	15	Balcony	15.46	13
Planning	9	Urban and rural	8.51	8	Social distance	15	Commemorate	15.4	16
Elastic	9	Office	8.47	6	Square	15	Synthesis	15.34	6
Sharing	9	Elastic	8.33	9	Sharing	15	Facade	14.72	5
Complex system	9	Basic needs	8.26	3	Open	14	Toughness	14.36	19
Originality	8	Public transit	8.26	3	Transport	14	Infrastructure	13.75	10
Emergency	8	Dynamic response	8.26	3	Balcony	13	Chinese tradition	12.89	5
Solution	8	Shelter space	8.26	2	Courtyard	12	Spatial folding	12.5	12
Significant contribution	8	Outdoor space	8.26	2	Commemorate	12	Pandemic resistance	11.49	7
High-rise buildings	6	Security	8.1	29	Park	12	Life module	10.75	3
Social distance	6	Population mobility	8.07	4	Wisdom	12	Streamline	10.69	25

Freq., frequency.

**Figure 2:**
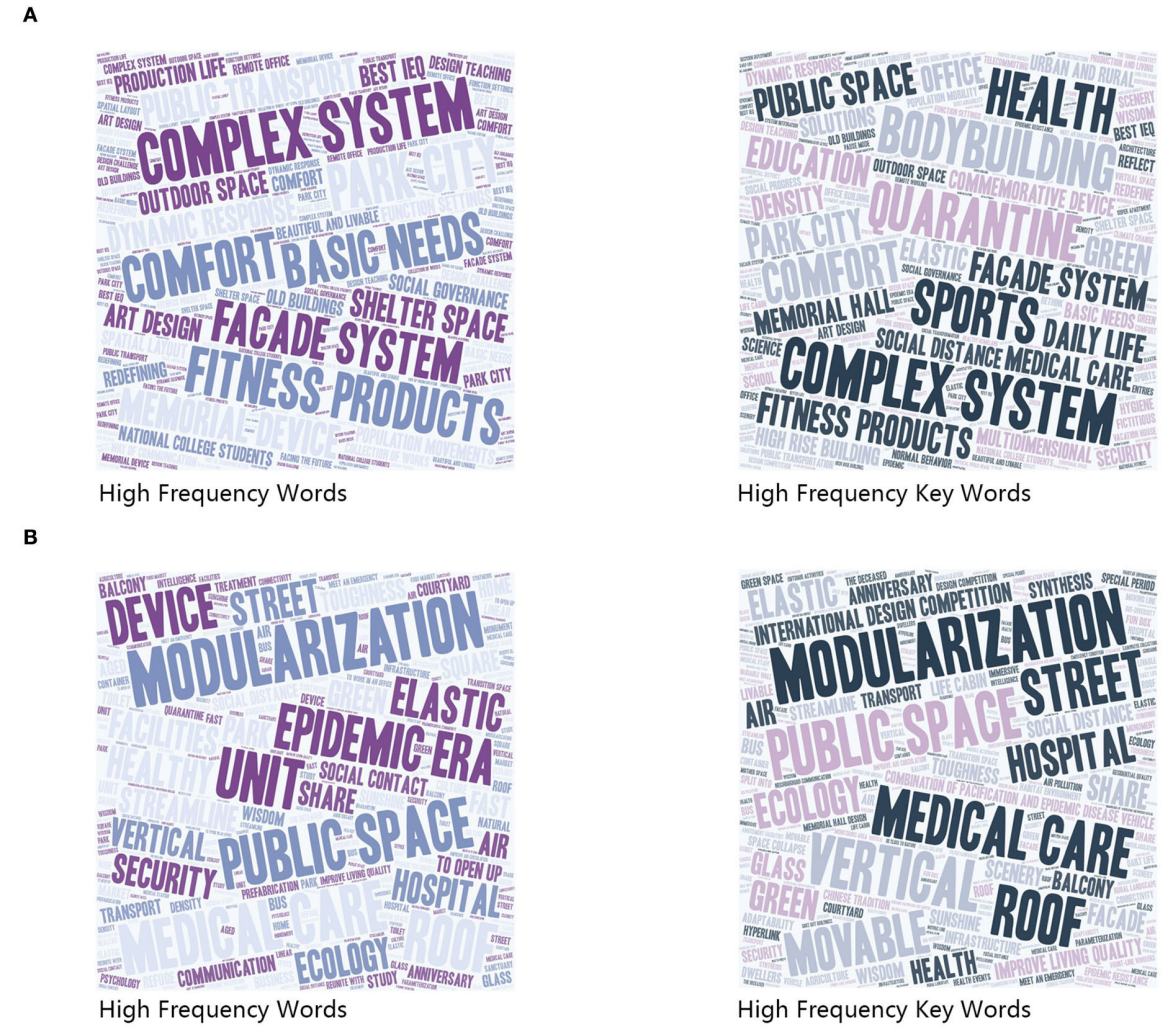
Competition themes and works high frequency words word cloud. **(A)** The themes word cloud. **(B)** The words word cloud.

The visualization results indicated that the parties designating the competition themes emphasized the pursuit of a healthy life (Figure 2). “Complex system,” “fitness products,” and “comfort” appeared multiple times in the list of new words, whereas “fitness,” “health,” “sports,” and “isolation” featured frequently among the keywords. Among the single words, the word frequencies of “community” and “health” had greater weights. The discussions of space presented diversified and cutting-edge designs among the drawing works information, concerned issues such as “healthcare,” “flow lines,” “social,” “infrastructure,” etc., buildings and spaces such as “community,” “hospital,” “public space,” “square,” “roof,” etc., resorted to strategies such as “modularization,” “elasticity,” “resilience,” “mobility,” “prefabrication,” “improving living quality,” “intelligence,” etc. All these reflected the competitors' positive thinking during the unusual period. Where modularity appears most frequently as a strategy.

### Results of the descriptive statistics

#### Results of frequency analysis

**Figure 3:**
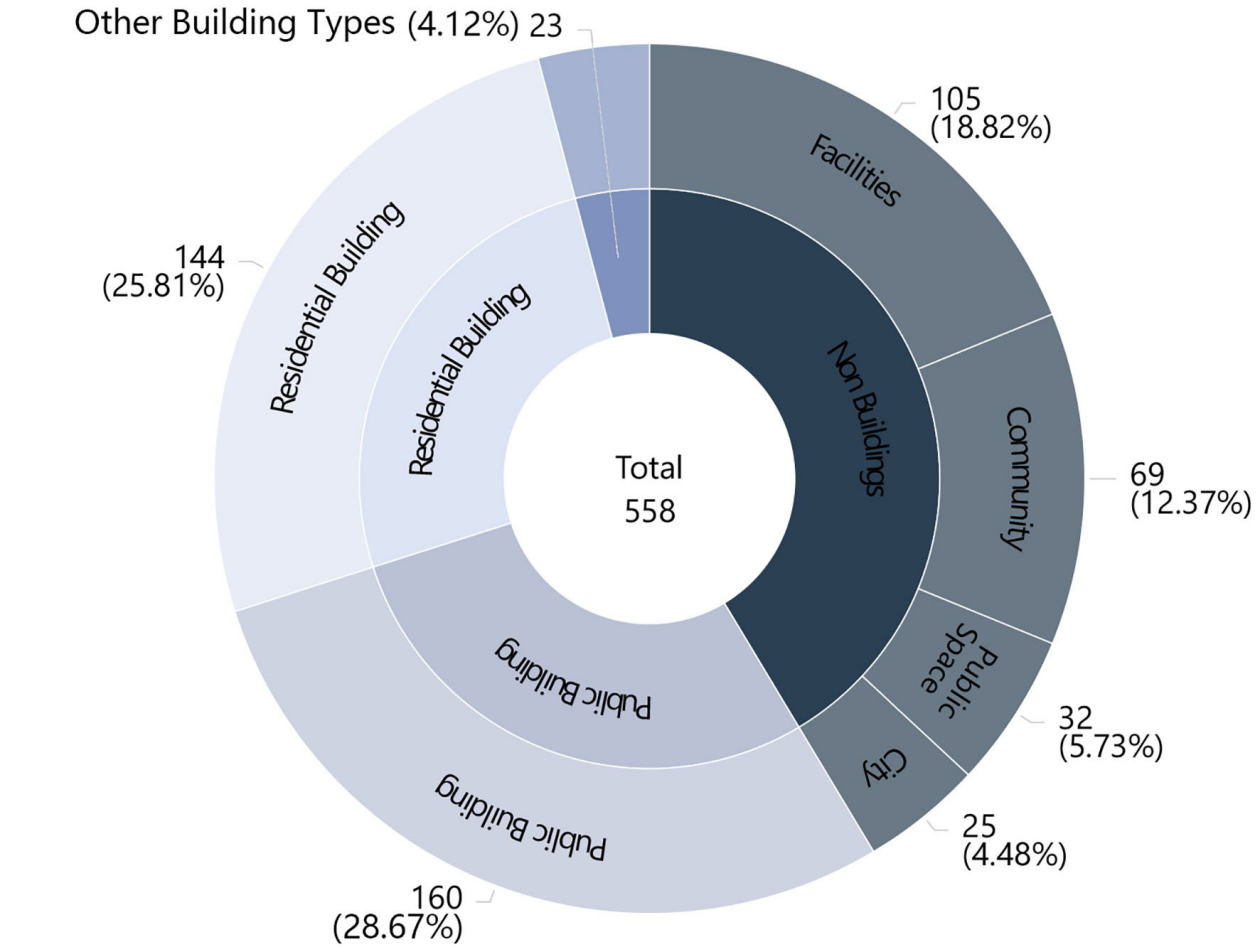
Distribution of building types.

**Figure 4:**
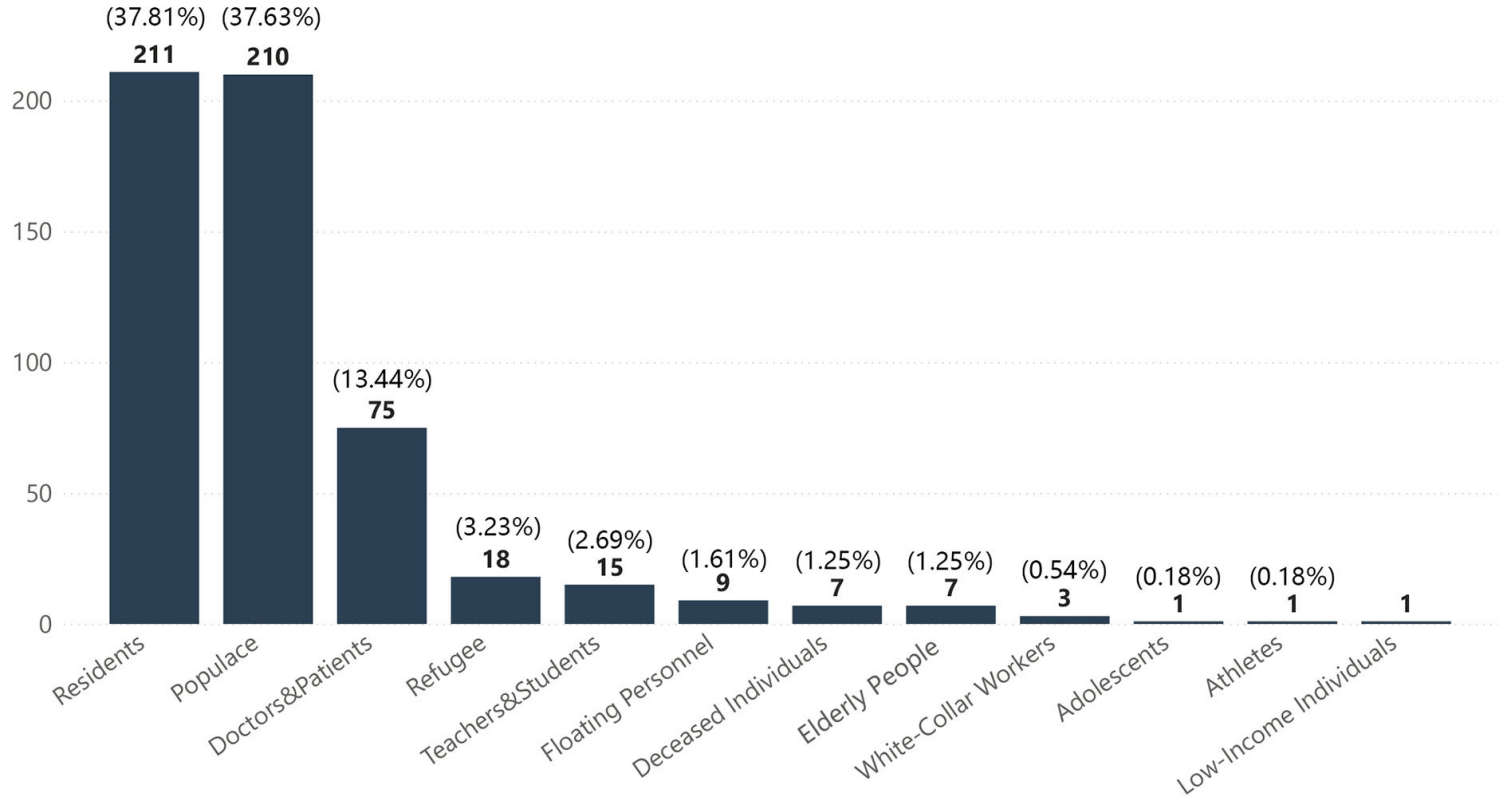
Distribution of target groups.

There was a wide range of competition works dealing with architectural/design carriers, with the scale ranging from the macroscopic level of cities to the microscopic level of facilities. These could be grouped into seven categories (Figure 3), with public buildings occupying the largest proportion at 28.7%. The other categories were residential buildings (26%), facilities (18.8%), communities (12.2%), public spaces (5.7%), cities (4.5%), other building types (4.1%), and works at the non-building level accounted for 41.4% of the total. Works dealing with the population were divided into 12 categories; the populace and residents categories accounted for 37.6 and 37.8% of the total, respectively (Figure 4). Doctors and patients accounted for 13.4%, and the remaining categories each accounted for ~3% or less of the total: refugees (3.2%), teachers and students (2.7%), floating personnel (1.6%), elderly adults (1.3%), deceased individuals (1.3%), white-collar workers (0.5%), low-income individuals (0.2%), adolescents (0.2%), and athletes (0.2%).

**Figure 5:**
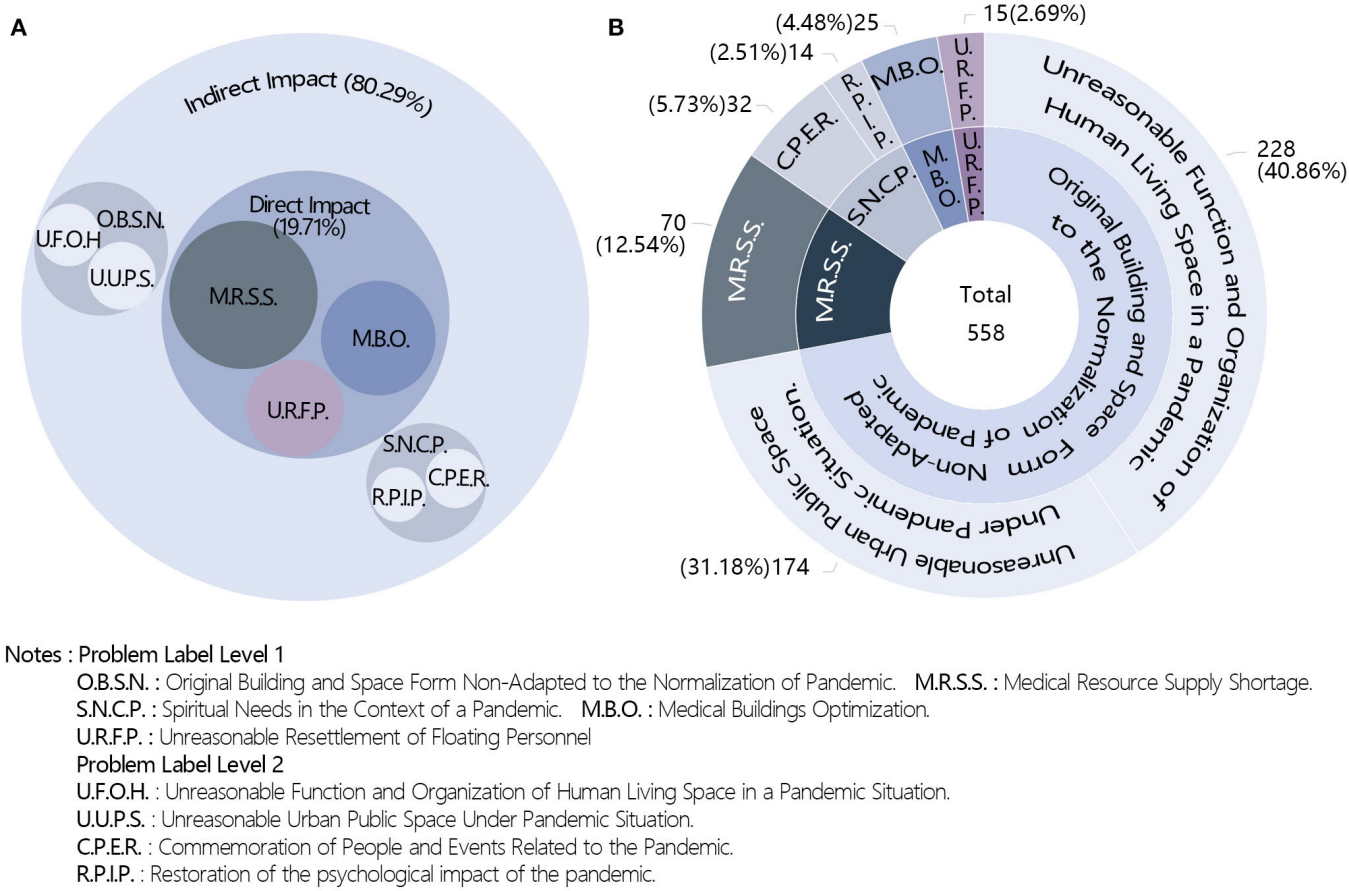
Distribution of targeted problems.

The target problems could be broadly divided into direct and indirect impacts of the pandemic (Figure 5A). The indirect impacts accounted for 80.2% of the total; 72.2% were concerns over the original building and space form non-adapted to the normalization of the pandemic situation. Unreasonable function and organization of human living space in a pandemic situation and unreasonable urban public space in a pandemic situation accounted for 40.9 and 31.2% of the total, respectively. The proportion of works focusing on spiritual needs in the context of a pandemic situation was 8.2%, and those for the commemoration of people and events related to the pandemic and restoration of the psychological impact of the pandemic were 5.7 and 2.5%, respectively. The proportion of works on the direct impacts of the pandemic was 19.8%, with the medical category receiving the most attention (17%). The medical resource supply shortage, medical building optimization, and unreasonable resettlement of floating personnel accounted for 12.5, 4.5, and 2.7% of the total, respectively (Figure 5B).

**Figure 6:**
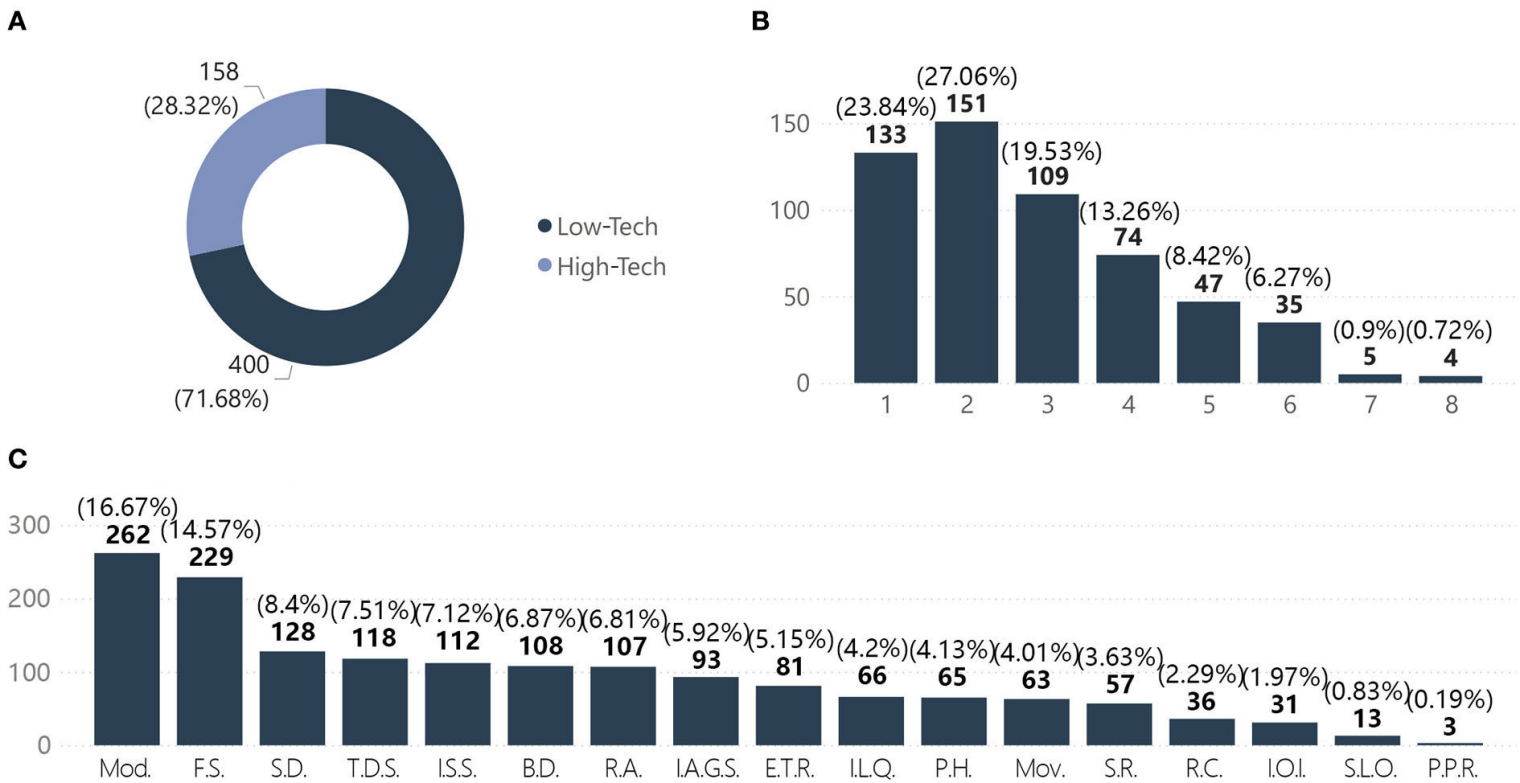
Distribution of strategies. **(A)** Technical orientation. **(B)** Quantities of strategies. **(C)** Strategies categories.

Statistics on technical orientation were used to examine whether the solutions envisaged in the face of the pandemic were high- or low-tech to better understand their orientation. High-tech solutions referred to the use of technologies and even fantastical methods, such as information intervention, construction of virtual worlds, and applications of advanced technologies. Low-tech solutions were based on existing or routine methods. Considering that the pandemic itself is a public health event, interventions in the built environment were often physical, resulting in a relatively high proportion of low-tech solutions for real life. After analyzing the samples, the proportions of low- and high-tech solutions were 71.7 and 28.3%, respectively (Figure 6A).

The quantity of strategies applied in the works ranged from 1 to 8, with the highest number of works (151, or 27.1% of the total) having applied two strategies. Only four works (0.7%) applied eight strategies. The mean and median of the quantity of strategies were 2.81 and 2, respectively (Figure 6B). Modularization and flexible space were the strategies that appeared the most frequently, at 262 and 229 times, respectively. These two strategies were adopted by almost half of the works, with 116 works (20.8%) adopting both strategies concurrently, making them the most recommended solutions by designers (Figure 6C). These various solutions highlighted the strategic directions being considered, which included adaptability of space, attention to social distancing, discussions on public vs. private spaces, immediate methods to solve needs, and spiritual needs. Notably, the application of building digitalization accounted for 20% of all the works. The pandemic has provoked designers to engage in in-depth thinking over the application of digital resources to solve real problems or even replace partial realities.

#### Results of cross-analysis

**Figure 7:**
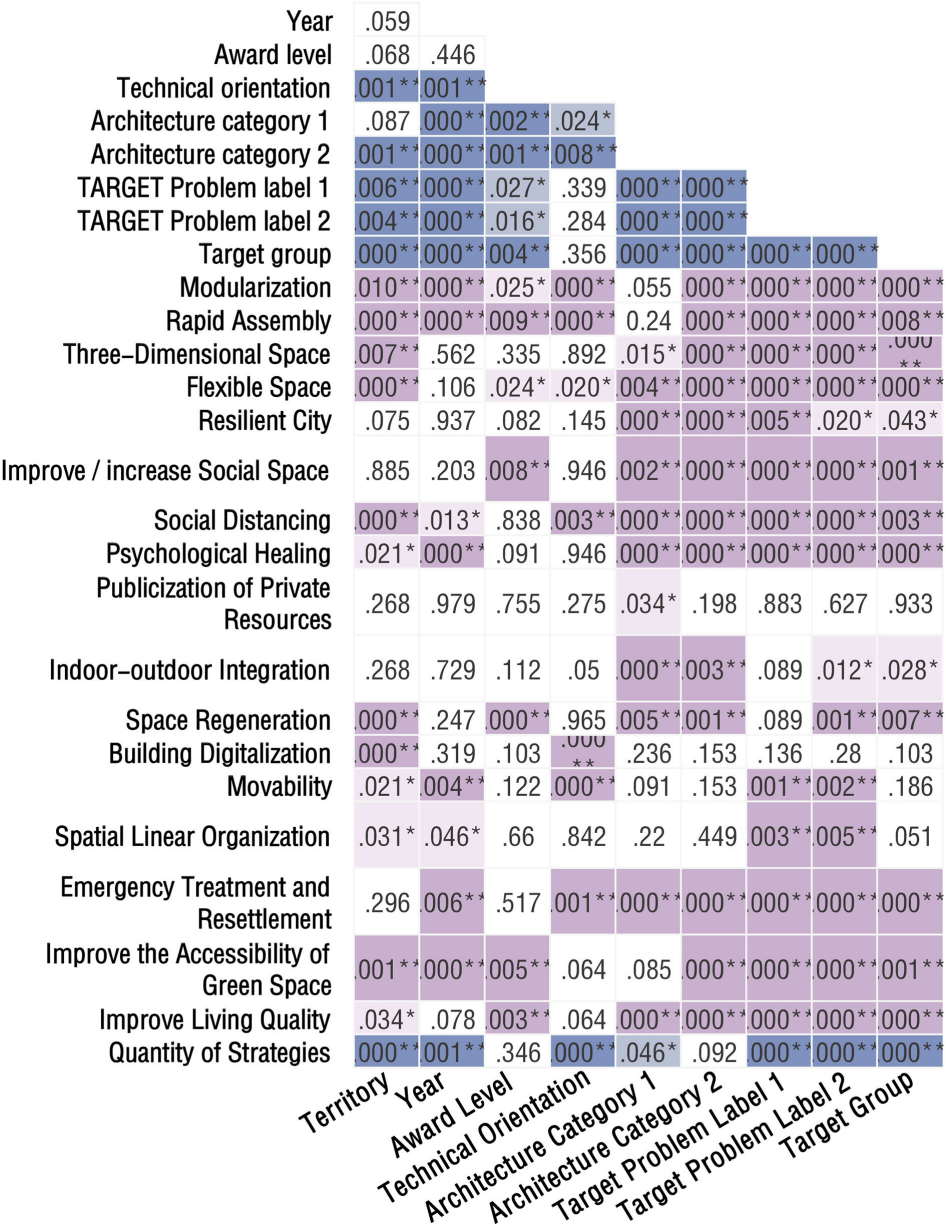
Pairwise cross-tabulation analysis results.

After the chi-square test, a pairwise test of the 11 variables was conducted. The test results for the strategic variables are shown in Figure 7. Next, cross-analysis was carried out between those variables that presented significance, and a comparative analysis of the differences was performed using the percentage as the main object.

##### Cross-analysis targeting the “territory” variable

**Figure 8:**
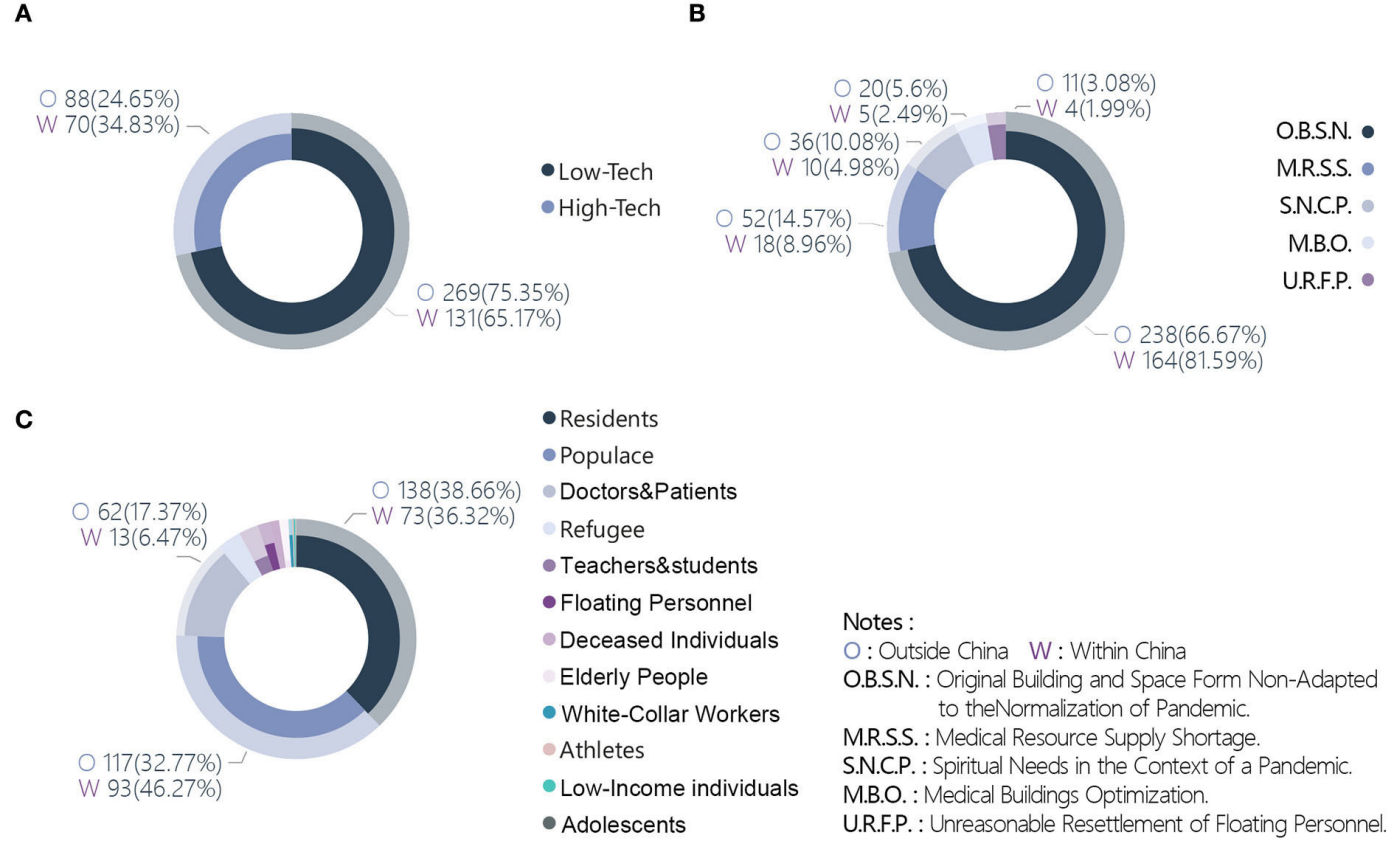
Cross-tabulation results with territory. **(A)** Territory and technical orientation. **(B)** Territory and target problem. **(C)** Territory and target group.

Six variables were significant for the “Territory” variable: technical orientation (*p* = 0.01), architecture category 2 (*p* < 0.001), target problem label level 1 (*p* = 0.006), target problem label level 2 (*p* = 0.004), target group (*p* < 0.001), and quantity of strategies (*p* < 0.001). The proportion of works that selected high-tech solutions was significantly higher within China than outside China (34.83 vs. 24.65%), meaning that more competitors within China chose high-tech solutions to handle the pandemic (Figure 8A). Similarly, the proportion of works that focused on public buildings and communities was significantly higher within China than outside China (33.83 vs. 25.77%, 18.41 vs. 8.68%). There were significantly more works from outside China than within China that focused on residential buildings (29.13 vs. 20.40%).

For the target problem, 81.59% of works from within China selected original building and space form non-adapted to the normalization of pandemics, which was significantly higher than the proportion of works from outside China (66.67%). By comparison, works from outside China focused on the medical resource supply shortage, the proportion of which was significantly higher than that of works within China (14.57 vs. 8.96%) (Figure 8B). For the target group, 46.27% of works from within China selected the populace, which was significantly higher than the proportion of works from outside China (32.77%). However, the proportion of the latter choosing doctors and patients was significantly higher than that of works from within China (17.37 vs. 6.47%) (Figure 8C).

**Figure 9:**
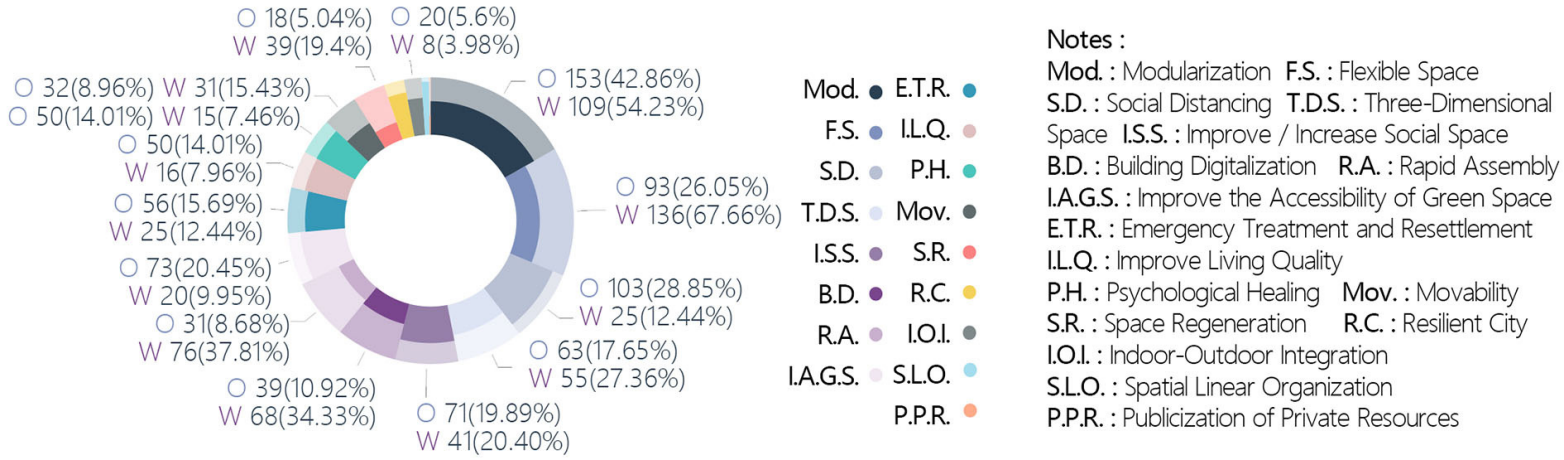
Cross-tabulation results with territory and strategies.

After analyzing the quantity of strategies, the proportions of works from outside and within China that selected a single strategy were 29.69 and 13.43%, respectively. The former region had a greater tendency to use one strategy to solve problems, whereas the latter had a higher proportion of works that applied a combination of methods to solve problems. In terms of specific strategies, works from within China had significantly more applications of the following strategies compared with works from outside China: modularization (54.23 vs. 42.86%), rapid assembly (37.8 vs. 8.68%), three-dimensional space (67.66 vs. 17.65%), flexible space (67.66 vs. 26.05%), space regeneration (19.40 vs. 5.04%), building digitalization (34.33 vs. 10.92%), and movability (15.42 vs. 8.96%). Works from outside China had significantly more applications of the following strategies compared with works from within China: social distancing (28.85 vs. 12.44%), psychological healing (14.01 vs. 7.46%), spatial linear organization (3.36 vs. 0.5%), improving the accessibility of green space (20.45 vs. 9.95%), and improving living quality (14.01 vs. 7.96%) (Figure 9).

##### Cross-analysis targeting the “year” variable

**Figure 10:**
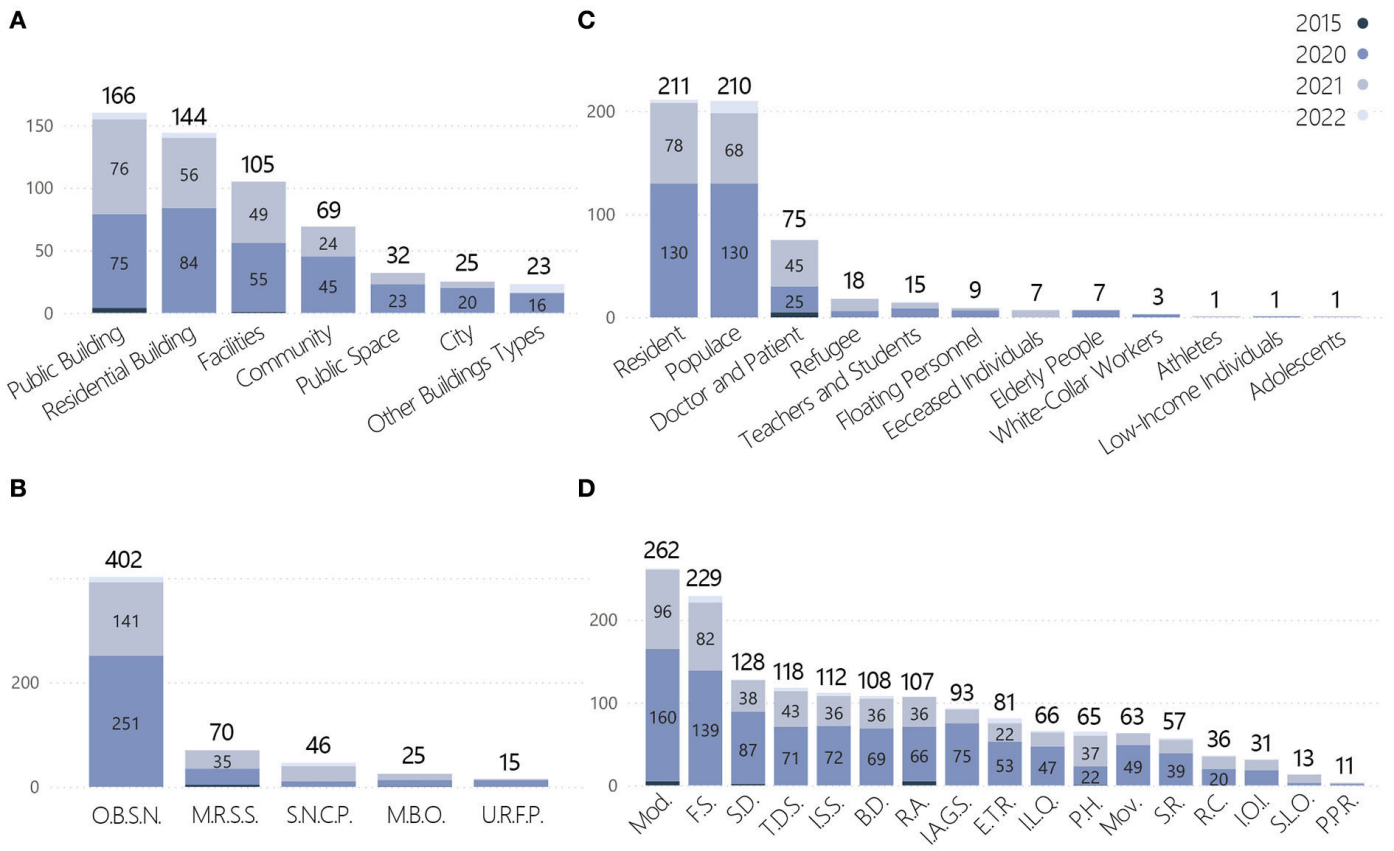
Cross-tabulation results with year. **(A)** Year and architecture category. **(B)** Year and target problem. **(C)** Year and target group. **(D)** Year and strategies.

The following variables were significant for the “Year” variable: technical orientation (*p* = 0.001), architecture category (*p* < 0.001), target problem label levels 1 & 2 (*p* < 0.001), target group (*p* < 0.001), and quantity of strategies (*p* = 0.001). In 2020, the proportion of works using high-tech solutions was 34.59%, which was significantly higher than the mean of 28.32%. In 2021 and 2022, the proportion decreased to 20.1 and 12.5%, respectively. For architecture category, the proportion of other buildings (parks, gates, bridges, monumental buildings, and structures) in 2022 was significantly higher than the mean (43.75 vs. 4.12%) (Figure 10A). In the 3-year period from 2020 to 2022, the proportions for the original building and space form non-adapted to the normalization of the pandemic, which was under the target problem label level 1, decreased annually from 78.9 to 64.4% and then to 62.5%. Over the same period, explorations of spiritual needs in the context of a pandemic situation were 3.5, 13.2, and 37.5%, respectively, increasing annually (Figure 10B). In 2022, 75.00% of the target group selected populace, which was significantly higher than the mean of 37.63%. In 2021, the proportion of doctors and patients as the target group was 20.55%, which was significantly higher than the mean of 13.44%. By comparison, the proportion of works targeting doctors and patients in 2015 was as high as 100%. The proportion increased from 7.9% to 20.5% in the period 2020–2022 (Figure 10C).

The modularization strategy was used by 50.31 and 43.84% of works in 2020 and 2021, respectively, but dropped sharply to 6.25% in 2022. The situation was similar for rapid assembly, with proportions of 20.75, 16.44, and 0% in 2020, 2021, and 2022, respectively. As the most basic and effective strategy for pandemic prevention and control, the yearly distribution of works on social distancing was consistent with that of the two aforementioned strategies: 27.36% in 2020, 17.35% in 2021, and only 6.25% in 2022. Over the same period, the use of the following two strategies similarly decreased annually: movability (15.41, 6.39, and 0%) and improve the accessibility of green space (23.58, 7.76%, and 6.25). In contrast, the use of psychological healing as a strategy increased annually at 6.92, 16.89, and 31.25%, respectively (Figure 10D).

A comparison was made between the competition works submitted in 2015 and those during the COVID-19 period. One hundred percent of works used the strategies of modularization and rapid assembly in 2015, 40% used social distancing, and 20% used psychological healing. These were all significantly higher than the mean. However, the following strategies, which occupied relatively high proportions subsequently, were not recorded: flexible space (41.04%), three-dimensional space (21.15%), improve or increase social space (20.07%), and building digitalization (19.35%).

##### Cross-analysis targeting the “target problem” variable

**Figure 11:**
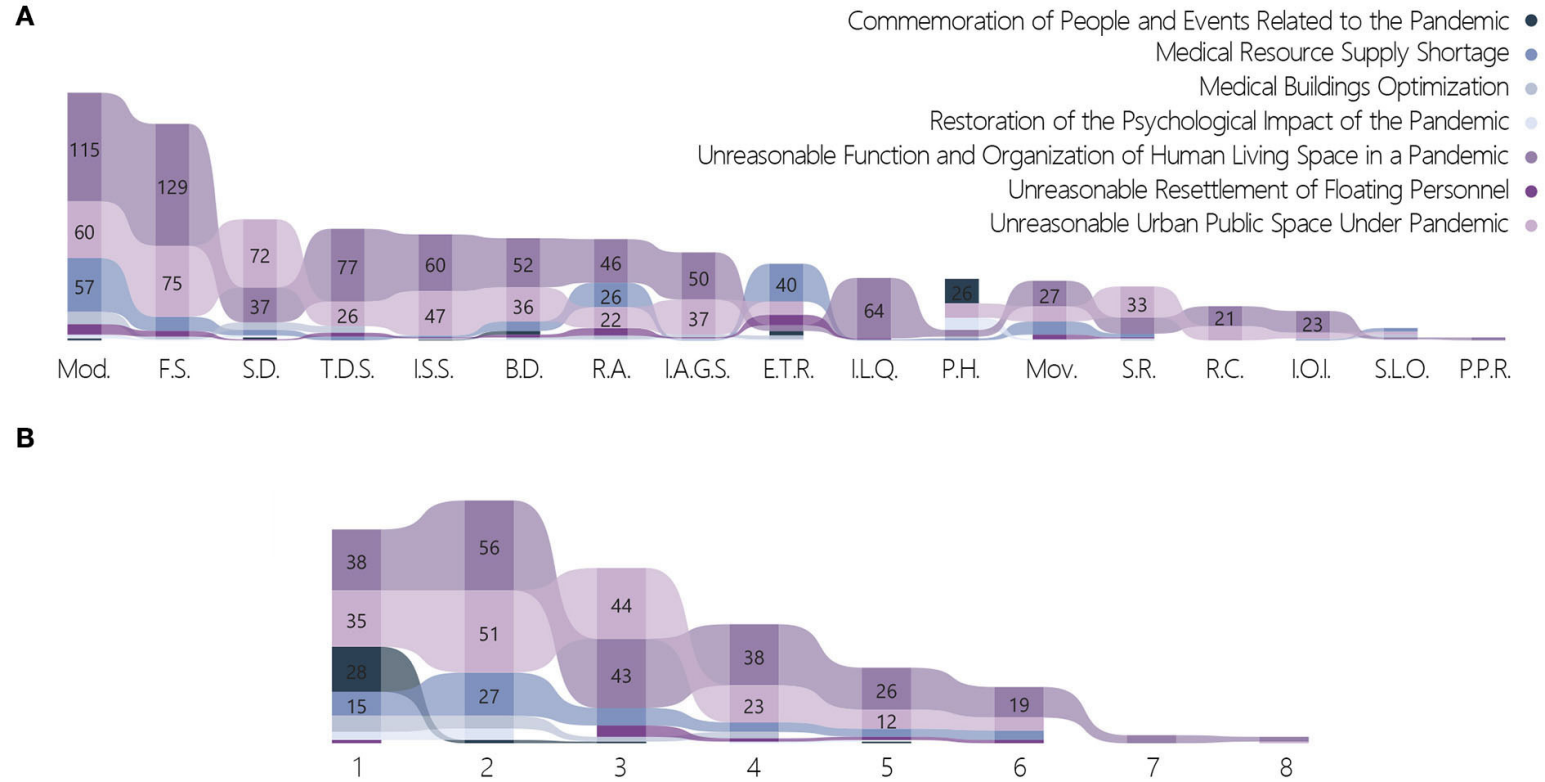
Cross-tabulation results with target problem. **(A)** Target problem and strategies. **(B)** Target problem and target problem label level.

Target problem label levels 1 & 2 were significant for the target group (*p* < 0.001) and quantity of strategies (*p* < 0.001). The characteristics of emergency needs during the pandemic being prominent were revealed in the cross-analysis of strategies and target problems. To address the issues of medical resource supply shortage and unreasonable resettlement of floating personnel, the proportions of strategies involving modularization (81.43, 73.33%), rapid assembly (37.14, 53.33%), movability (20.00, 33.33%), and emergency treatment and resettlement (57.14, 73.33%) were significantly higher than the means (46.95, 19.18, 11.29, and 14.52%, respectively). The use of above strategies reflected the need for flexible emergency functions when dealing with the issues of provision of medical care and temporary resettlement. For the issues of the original building and space form non-adapted to the normalization of a pandemic situation and medical building optimization, applications of the social distancing strategy (27.11, 32.00%) were also significantly higher than the mean of 22.94% (Figure 11A).

For medical resource supply shortage and medical building optimization, the proportions of works applying the spatial linear organization strategy and related to medical problems (5.71, 12.00%) were significantly higher than the mean (2.33%). These reflected the importance of effective solutions for streamlining in medical issues. The proportions of works under spiritual needs in the context of the pandemic situation and medical building optimization that selected a single strategy were 71.74 and 40.00%, respectively. This was significantly higher than the mean of 23.84%. For commemoration of people and events related to the pandemic, which was under spiritual needs, the proportion of works choosing a single strategy was high and reached 87.50%. This was significantly higher than the mean of 23.84% (Figure 11B). Comparing the research results with other problems illustrated that issues related to spiritual needs and medical buildings were mostly addressed using a single strategy, whereas complex strategies were required to solve conflicts in the supply of medical resources and unreasonable functional organization.

##### Cross-analysis targeting the “target group” variable

**Figure 12:**
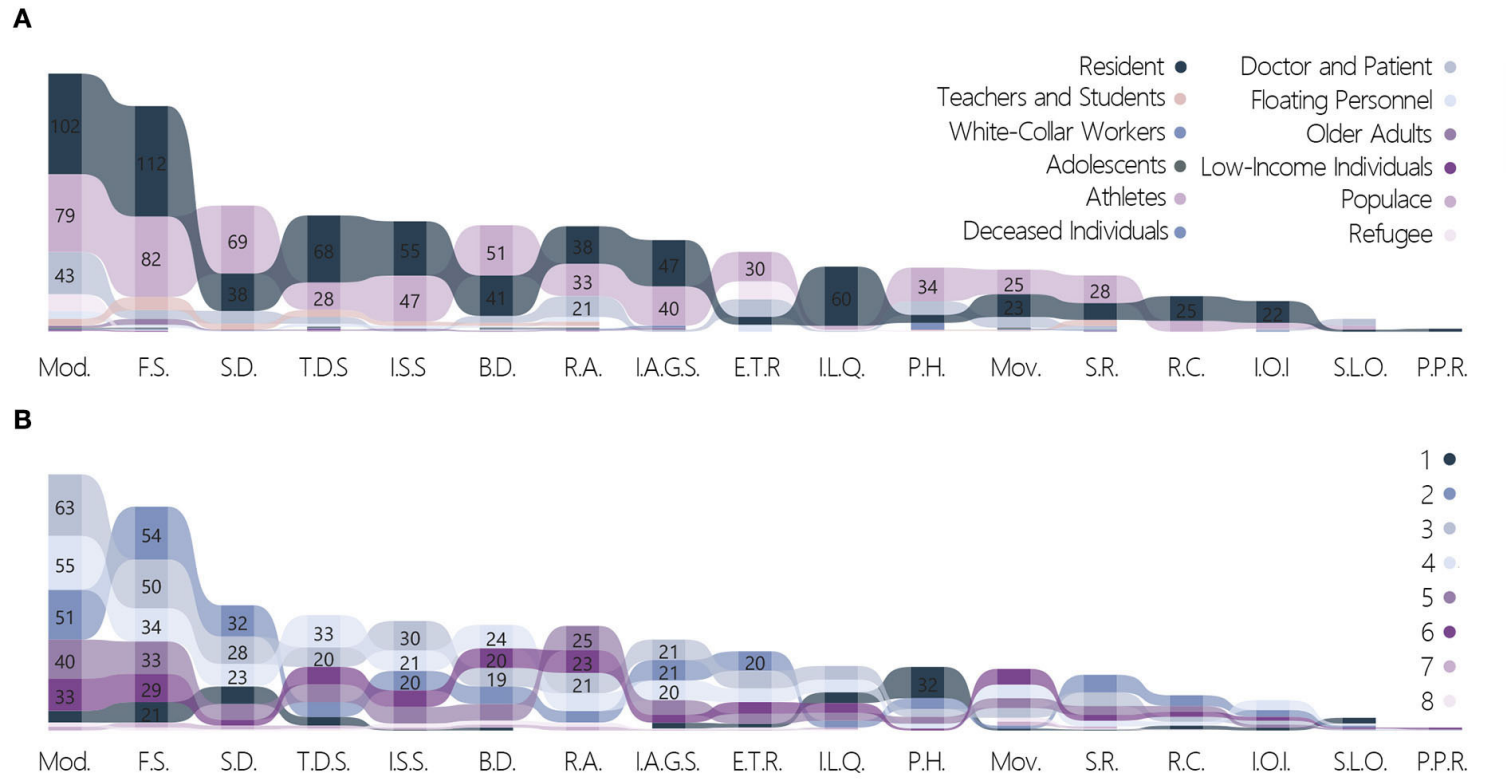
Cross-tabulation results with target group & quantity of strategies and strategies. **(A)** Target group and strategies. **(B)** Quantity of strategies and strategies.

The cross-analysis of the target groups and strategies showed that (Figure 12A) only one strategy was selected in all works dealing with deceased individuals. The proportion of works that chose four strategies to manage teachers and students was 26.67%, which was significantly higher than the mean of 13.26%. The countermeasures at the spiritual level often applied a single strategy; the combination of multiple strategies was often considered for teachers and students, who constituted a complex and diverse group.

The percentages of strategies used for different groups were analyzed, and several conclusions were arrived at after comparing the average proportions of each item. First, all strategies were included for use on the populace, which is the most general sense of a group. Social distancing accounted for 53.08% of the total, which was significantly higher than the mean of 41.04%. This has also been proven to be an effective strategy for pandemic prevention and control worldwide.

All strategies were similarly included for residents as a group. The application rates of five strategies—three-dimensional space, flexible space, indoor–outdoor integration, improving the accessibility of green space, and improving living quality—were significantly higher than the mean. These strategies were approaches that targeted the living conditions of residents to deal with the pandemic environment. For the four strategies of modularization, rapid assembly, psychological healing, and emergency treatment and resettlement, the focus on doctors and patients was significantly higher than the mean. This was mainly reflected in responses to the instantaneous surge in the demand for medical resources and psychological relief.

##### Cross-analysis targeting the other labels variable

The “Quantity of strategies” variable showed that, works on psychological healing constituted the highest number with only one strategy applied, amounting to 32 out of 133 works. This was followed by flexible space, which appeared in 21 works (Figure 12B). When multiple strategies were combined for application in one work, the following 11 strategies were often grouped together: modularization, rapid assembly, three-dimensional space, flexible space, improving/increasing social space, space regeneration, building digitalization, movability, emergency treatment and resettlement, improving the accessibility of green space, and improving living quality. All works applying eight strategies used the following five strategies: modularization, rapid assembly, improving/increasing social space, building digitalization, and movability.

**Figure 13:**
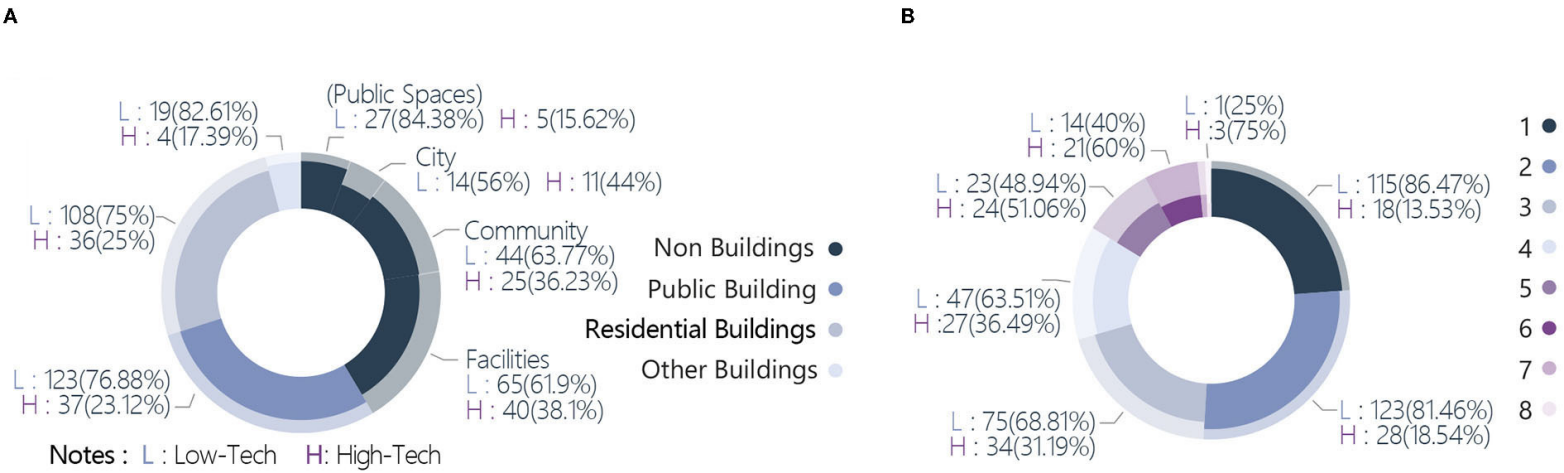
Cross-tabulation results with technical orientation. **(A)** Technical orientation and architecture category. **(B)** Technical orientation and quantity of strategies.

The “Technical orientation” variable was significant in architecture categories 1 & 2 (*p* = 0.024, *p* = 0.008) and quantity of strategies (*p* < 0.001). In the architecture category, the proportion of works on non-buildings with high-tech solutions was 35.06%, which was significantly higher than the mean of 28.32%. Under the secondary label, the proportions of works on the city (44.00%) and facilities (38.10%) were significantly higher than the mean of 28.32%. The proportion of works on public spaces and public buildings with low-tech solutions was significantly higher than the mean (84.38 & 76.88% vs. 71.68%) (Figure 13A). These statistics indicated that high-tech solutions are more applied to the categories of cities and facilities, whereas more low-tech solutions are proposed for public buildings.

In the cross-analysis between quantity of strategies and technical orientation, the proportions of works that incorporated one and two strategies involving low-tech solutions were 86.47 and 81.46%, respectively, significantly higher than the mean of 71.68%. The proportions of works using six and eight strategies and involving high-tech solutions were 60.00 and 75.00%, respectively, significantly higher than the mean of 28.32% (Figure 13B). The implication was that the lower the number of strategies used, the higher the tendency to use low-tech solutions, and the higher the number of strategies used in the works, the more likely that these strategies contained high-tech solutions.

**Figure 14:**
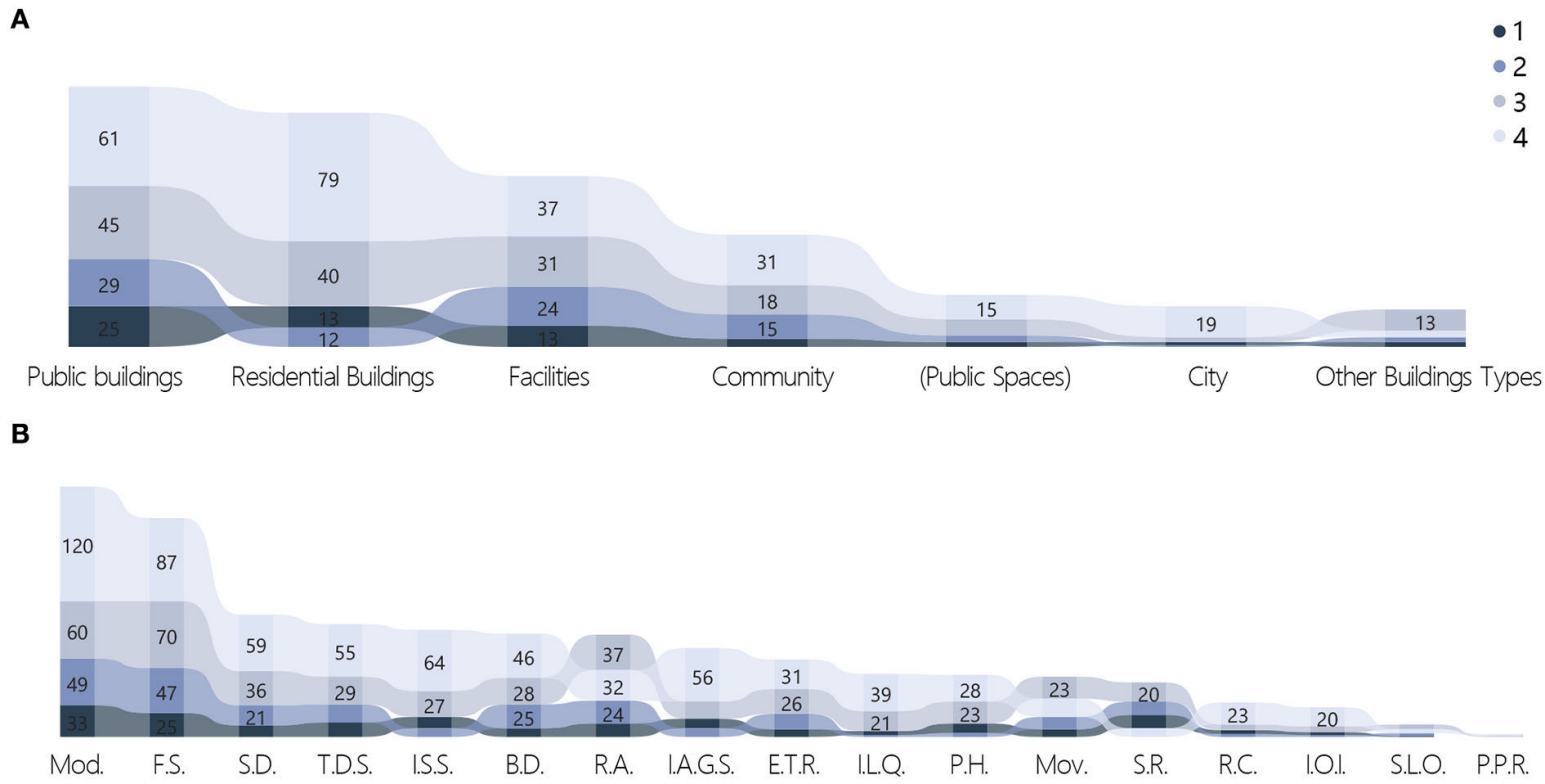
Cross-tabulation results with award level. **(A)** Award level and architecture category. **(B)** Award level and strategies.

The following variables were significant for the “Award level” variable: architecture categories 1 & 2 (*p* = 0.002, *p* = 0.001), target problem labels 1 & 2 (*p* = 0.027, *p* = 0.016), technical orientation (*p* < 0.001) and target group (*p* = 0.004). Winning works related to public buildings or facilities did not necessarily have more options to choose from within the buildings category. For example, 39.06% of works with Level 1 awards covered public buildings, which was significantly higher than the mean of 28.67%. For Level 2 awards, 50.00% of the works dealt with non-buildings, of which the proportion that selected facilities was 27.27% and the mean was 18.82%. In the selection of issues to respond to, the winning works emphasized the issue of contrast of medical resource supply; it was the focus of 18.75% of works granted Level 1 awards, which was higher than the mean of 12.54%.There was a significant difference between award levels for the strategy of space regeneration. The proportions of works with awards at Levels 1 and 2 that adopted this strategy were 21.88 and 15.91%, respectively. These were significantly higher than the mean of 10.22%. By comparison, only 3.66% of works with Level 4 awards applied this strategy (Figure 14).

## Discussion

### Focus

There were equal numbers of works dealing with the populace and residents, jointly accounting for 75.4% of the total. Most works addressing people affected by the pandemic chose to cover a broad scope. Among them, nearly 40% focused on residents, clearly revealing a perception of the vast impact of the pandemic on daily living. The proportion of works for doctors and patients was significantly higher for other occupations/groups of people, and this group was also the most directly affected by the pandemic. Other groups—teachers and students, white-collar workers, and athletes—accounted for a relatively small proportion from the occupational perspective. Attention was paid to elderly people and adolescents as members of vulnerable age groups. Other originally vulnerable groups in society, such as refugees, the poor, and floating personnel, combined to form the subjects of 5% of the total works. At the same time pandemic also redefines the vulnerability population (43).

Secondary health impacts include economic stability, education, healthcare, neighborhood and built environment, and social and community contextual factors (44). The indirect impacts of the pandemic on life constituted the main issue of focus, accounting for 80.2% of the total. Incompatibility with the physical space was a primary consideration. The concern over issues related to people's living spaces was greater than that for public spaces, and living space issues were also the biggest problems experienced by the base population during the pandemic. Attention to spiritual needs also exceeded 8%. These showed that, although issues with the physical space were predominant during the pandemic, impacts at the spiritual level were also great and could not be ignored. Further evidence of the dramatic psychological impact of COVID-19 and the urgency for intervention (45–47). The direct impacts of the pandemic were relatively concentrated, with the greatest emphasis being on medical care. The most prominent problem was medical resource supply, which was related to the critical demand placed on the medical system for pandemic control. Unreasonable resettlement of floating personnel was also a concern for the vulnerable populations in society.

Among the strategies used in the studied works, modularization and flexible space were the most popular, applied in nearly half of the works. The proportion of works with simultaneous applications of both strategies was also high. For the statistics on text mining, modularization was also the most mentioned strategy in terms of word frequencies and keywords. This highlighted the consensus among designers to be flexible when adapting to the uncertainties brought about by the pandemic. Social distancing has been widely discussed in Pandemic Influenza (48, 49), and simulation validation of targeted social distancing design has been performed (50). Improving/increasing social space and social distancing seemed to be a pair of mutually exclusive strategies (51, 52), which implied the dilemma that the pandemic created for people: they not only had to maintain a social distance for effective pandemic prevention and control but also had to improve social health through interactions (53, 54). A variety of solutions to this dilemma were provided by these works through their specific forms of realization.

### Differences

Among the collected works, those from within China accounted for nearly 40% of the total. China was the first country to report cases of COVID-19 infections. In the subsequent 3 years, the government imposed the general policy of dynamic eradication of infections to deal with the pandemic. The similarities and differences between the situations within and outside China could be examined by comparing the works from both regions. From the perspective of the building types involved, works from within China during the pandemic paid more attention to meso- and community-level issues and those related to public buildings. Works from outside China focused on issues related to residential buildings. For issues related to the pandemic, works from within China emphasized the indirect impacts, whereas those from outside China were more interested in the problems with the medical system that were exposed by the pandemic.

In parallel, there was a contrast between works from within and outside China in terms of the target groups: the former included more works dealing with broad scopes, such as the populace, whereas the latter focused on doctors and patients, who were the most closely associated with the pandemic. From the perspective of the strategies used, works from within China involved adjustments in the construction of physical space; works from outside China that applied strategies for the spiritual space accounted for a larger proportion. The proportion of works from outside China that applied social distancing was much larger than those from within China. As early as 100 years ago, during the 1918–19 influenza pandemic, the New York City Department of Health enforced several social distancing policies at the same time, including staggered business hours, compulsory isolation, and quarantine, which likely led to New York City suffering the lowest death rate from influenza on the eastern seaboard of the USA (55). In terms of the technical orientation, a greater proportion of works from within China chose high-tech solutions to deal with the pandemic.

### Trends

It has been nearly 3 years since the COVID-19 outbreak. The findings of the cross-analyzes relating to the year as a variable showed substantial variations, which led to evident trends in terms of the questions and groups of people that the works dealt with, as well as the use of strategies. Over the 3-year period of 2020–2022, the original building and space form non-adapted to the normalization of the pandemic constituted the largest proportion of works. The highest number was in 2020, when nearly 80% of the works discussed this issue. Although 60% of the works still focused on this issue in 2022, the proportion declined annually. In contrast, discussions of psychological needs during the pandemic increased annually from 3.5 to 37.5%. In the early stage of the pandemic, the issue of incompatibility with the physical space accounted for the absolute majority of works, and it was the most prominent and influential issue. As the pandemic progressed, its impacts on the spiritual level gradually emerged and received more attention. Correspondingly, there were more explorations of commemoration and restoration, both aspects of spiritual needs. The variable of people was initially dominated by the populace and residents, but both aspects declined in 2021. Instead, the focus on doctors and patients was on the rise.

The use of high-tech solutions in works decreased over the 3 years and was replaced by works with low-tech solutions. Strategies that focused on adaptability—modularization, rapid assembly, and movability—accounted for a high proportion of all works. However, their use showed a declining trend over the 3 years, as did use of strategies involving social distancing. After a comparison with the 2015 competition works, the core strategies adopted in the 2020–2022 works were found to be very similar and related to adaptations to emergencies and flexibility. Therefore, it was surmised that modularization, rapid assembly, movability, and social distancing were important strategies to rapidly address the changing needs in the early stage of the pandemic. However, discussions of these conventional strategies gradually declined with the progress of the pandemic. Instead, there was a shift to the discovery of and solutions to diverse secondary social problems. Notably, the strategy of building digitalization had consistently maintained a mean of nearly 20%. The pandemic had further promoted the space and form in which online activities occurred and also prompted people to make more extensive use of digital technologies to avoid pandemic risks. This would undoubtedly accelerate the development of related technologies such as digital twin (56, 57). In essence, the pandemic has pressed the fast-forward button on the growth of the “Metaverse.”

## Conclusion

To explore ways to intervene public health in the post-pandemic era and to provide a rethinking of the built environment, this study launched research on the issues and solutions presented by architectural design competitions and drawing works under the COVID-19 theme, as well as the variations in their characteristics and the evolutionary trend. The findings indicated that the direct impacts revolved around the medical system and temporary resettlement; the indirect impacts, which accounted for a relatively high proportion of works, were related to the incompatibility between the existing environment and spiritual needs. This corresponded with the broad scope in terms of the target groups of the works—namely, the populace and residents. These strengthened the perception of the extensive impacts that the pandemic has had on life. Flexible emergency adaptations featured predominantly among the strategies extracted from the works, among which modularization and flexible space were the most important solutions. Once again, the extensive use of modularization was in response to concerns about the incompatibility between the existing environment and emergency problems such as medical care and settlement of people affected, and it also highlighted the general trend of prefabricated buildings.

Cross-analysis of the works indicated multiple differences in terms of territory, target group, target problem, architecture category, technical orientation, and strategies, which produced a series of meaningful conclusions. Works from within China applied more high-tech solutions, paid more attention to the populace, and focused more on the impact of the pandemic on the people's lives; by comparison, works from outside China paid more attention to direct issues such as medical resource supply and spiritual needs. A single strategy was often used to deal with issues related to spiritual and medical needs, and these issues were more frequently discussed in works that won high-level awards.

After analyzing the changes with the passage of time, we found declining attention to issues pertaining to physical space but increasing attention to spiritual issues annually. The use of strategies involving emergency flexible adaptations decreased, but the proportion of strategies encompassing psychological healing increased. The application of high-tech solutions declined overall, but the application of building digitalization remained stable. These reflect the change in people's awareness, concerns, and reflections on the pandemic as it develops and evolves.

This pandemic presents a common challenge to all systems in society. Architectural design, as a critical undertaking of intervening in the built environment, should take the responsibility of actively combating the pandemic. The analysis of architectural design competitions under the theme of the pandemic enabled us to better understand its impacts on society in a more diverse way, leading to responses and suggestions within the field of construction that can deal with public health issues in the context of the pandemic and which are proposed in a more rapid and timely manner compared to the relatively lengthy design–build–use–evaluate cycle involved in evidence-based research.

### Implications

This research aimed to explore the patterns, differences, and trends of the themes and works submitted for architectural design competitions and to provide additional public health interventions and directions by rethinking the built environment. The impacts of the pandemic on society as a whole will be long-lasting and usher in permanent changes in the fields of public health and architectural design. In the early stage of the pandemic, social management and control methods were relied upon for effective prevention and control. This further confirmed that the pandemic not only affected the medical system but had impacts radiating to all aspects of society. The effectiveness of the environment at suppressing the pandemic was also clearly demonstrated. The field of public health gradually attracted attention as an intervention method. Considering that architectural design can provide additional methods with far-reaching influence, competition works based on the theme of public health should be treated as important knowledge resources for management and decision-making.

Architectural design is a creative process. There is often insufficient time for substantial preliminary research because of the engineering requirements. New problems arising from the impact of the pandemic require vast accumulation of experiences and thoughts. The targeted problems and strategies for solutions compiled in this study can provide perspectives and ideas for design practice. The myriad types of strategies and solutions form a valuable database of case studies, which will provide designers with comprehensive ideas for problem solving. Concurrently, city managers receive decision-making suggestions that prompt them to pay attention to the impact at all levels, organize a diverse design team, and consider the wellbeing of various groups of people.

### Limitations

Although as many competition entries as possible were sampled from the Internet in this study, the generalizability of the findings remains limited. First, the number of competitions and works compiled from 2022 was significantly lower than in the other years. This was because the samples were collected in the current year. More importantly, the relevant sample size had indeed shrunk significantly. This situation was most likely due to the long time span, during which the tracking of the pandemic and the attention of society and individuals varied with the changes in the pandemic itself. Design competitions typically revolve around the prevailing social focus receiving the most attention. The violent shock to society in the early stage of the pandemic led to the emergence of intensive thematic discussions in the past 2 years, resulting in a huge base number of works and a wide variety of perspectives.

The impacts of the pandemic have gradually become clear after in-depth discussion, such that the attention of organizers and contestants of design competition have gradually been diverted to other social issues. For example, several important series of competitions held in China in 2022 had begun discussing topics such as “urban connectivity,” “landscape folding,” and “connecting tradition and the future.” Such topics no longer led participants to the pandemic perspective.

At the same time, the use of only Chinese and English in our online searches omitted competition information posted in other languages and increased the proportion of works from within China. Finally, because competition works were adopted as the samples, there was no information on post-use evaluation after implementation of the proposals. Objectively, this was a limitation faced between balancing the prevailing urgent needs and the long cycle inherent in the implementation of architectural designs. Nevertheless, this study has provided the basis for follow-up verification and comparative research that we plan to undertake.

## Data availability statement

The original contributions presented in the study are included in the article/supplementary material, further inquiries can be directed to the corresponding author/s.

## Author contributions

PH contributed to conception and design of the study and wrote the first draft of the manuscript. LW, YS, and XZ organized the database. PH and YS performed the statistical analysis. LW drew the charts. All authors contributed to manuscript revision, read, and approved the submitted version.
